# Episodic Memory Dysfunction and Effective Connectivity in Adult Patients With Newly Diagnosed Nonlesional Temporal Lobe Epilepsy

**DOI:** 10.3389/fneur.2022.774532

**Published:** 2022-02-10

**Authors:** Aftab Bakhtiari, Agnes Balint Bjørke, Pål Gunnar Larsson, Ketil Berg Olsen, Marianne C. Johansen Nævra, Erik Taubøll, Kjell Heuser, Ylva Østby

**Affiliations:** ^1^Department of Psychology, Faculty of Social Sciences, University of Oslo, Oslo, Norway; ^2^Division of Clinical Neuroscience, Department of Neurology, Oslo University Hospital, Rikshospitalet, Oslo, Norway; ^3^Division of Neurology, Rheumatology and Habilitation, Department of Neurology, Drammen Hospital, Vestre Viken Hospital Trust, Drammen, Norway; ^4^Faculty of Medicine, Institute of Clinical Medicine, University of Oslo, Oslo, Norway; ^5^Section of Clinical Neurophysiology, Division of Clinical Neuroscience, Department of Neurosurgery, Oslo University Hospital–Rikshospitalet, Oslo, Norway

**Keywords:** autobiographical memory, electroencephalography, memory disorders, newly diagnosed epilepsy, hemispheric differences, temporal lobe epilepsy, network

## Abstract

**Objective:**

Epilepsy is associated with both changes in brain connectivity and memory function, usually studied in the chronic patients. The aim of this study was to explore the presence of connectivity alterations measured by EEG in the parietofrontal network in patients with temporal lobe epilepsy (TLE), and to examine episodic memory, at the time point of diagnosis.

**Methods:**

The parietofrontal network of newly diagnosed patients with TLE (*N* = 21) was assessed through electroencephalography (EEG) effective connectivity and compared with that of matched controls (*N* = 21). Furthermore, we assessed phenomenological aspects of episodic memory in both groups. Association between effective connectivity and episodic memory were assessed through correlation.

**Results:**

Patients with TLE displayed decreased episodic (*p* ≤ 0.001, *t* = −5.18) memory scores compared with controls at the time point of diagnosis. The patients showed a decreased right parietofrontal connectivity (*p* = 0.03, *F* = 4.94) compared with controls, and significantly weaker connectivity in their right compared with their left hemisphere (*p* = 0.008, *t* = −2.93). There were no significant associations between effective connectivity and episodic memory scores.

**Conclusions:**

We found changes in both memory function and connectivity at the time point of diagnosis, supporting the notion that TLE involves complex memory functions and brain networks beyond the seizure focus to strongly interconnected brain regions, already early in the disease course. Whether the observed connectivity changes can be interpreted as functionally important to the alterations in memory function, it remains speculative.

## Introduction

Temporal lobe epilepsy (TLE) is the most common type of focal epilepsies and is typically associated with structural and functional alterations of the temporal lobe. Recently, new etiological models have been proposed, indicating that epilepsy might be a network disorder whose effects and deterioration spread beyond the epileptogenic zone (EZ) to strongly interconnected regions (1–4). In support of this, studies in patients with TLE have explored network alterations in regions ipsi- and contralateral to the EZ, and also the default mode network (DMN) (1, 4–8). In general, these studies are conducted several years after patients are diagnosed with epilepsy, and indicate that the functional connectivity alterations are more extensive in the medial temporal lobe (MTL) regions ipsilateral to the EZ, whereas the inter and contralateral connectivity alterations seem to be more varying and diffuse (4, 8–10). The development of such network alterations need to be better understood, and investigations into the connectivity patterns in the newly diagnosed patients with TLE that might be the first step in this direction. Indeed, the brain consists of billions of neurons, which together constitute complex structural networks—and it is believed that such networks are what provide the basis of behavioral and cognitive processes in humans (11, 12). By measuring EEG connectivity, we can directly assess these networks, and investigate their association with behavior and cognition.

Another source of evidence suggesting that TLE might involve network disturbances is the way memory may be affected in some patients. Traditionally, memory is investigated using standardized neuropsychological list-learning tests (13), whose ecological validity regarding everyday reports of autobiographical episodic memory complaints has been questioned (14). Autobiographical, episodic memories differ from modality-specific list-learning in their phenomenological qualities, typically described in the terms of vividness, perceptual details, reliving of the past, and cohesiveness of a narrative (15), and patients with TLE might typically report this as problems with remembering things they did in the past (16).

Although episodical, autobiographical memory is dependent on hippocampal integrity, it is also subserved by diverse brain regions, most notably regions involved in the default mode network—including parietal and medial prefrontal regions (17, 18). Episodic memory retrieval is considered a constructive process where bits and pieces of information are merged in order to recreate both past and future events (17). This allows for a flexible extraction, recombination, and reassembling of elements to remember past events, and to simulate future ones (19, 20). So while the key structures of episodic memory consolidation may be located in the MTLs, extratemporal regions are likely to be involved in the constructive processes necessary for episodic memory retrieval (17).

In further support of this, several studies have found the parietal cortex to be involved in episodic memory tasks (21–25). Interestingly, the lateral parietal cortex has direct anatomical connections with several key brain areas, such as the dorsolateral prefrontal cortex and the temporal cortex, and has reciprocal connections with structures in the MTLs, such as the entorhinal, parahippocampal, and hippocampal regions (23). It has, therefore, been implied that parietal regions may play an important role in the retrieval of personal memories, especially for the reconstructive processes that are necessary for the episodic memory. In this study, we investigate the hypothesis that TLE may constitute a wider network disorder already in its early stages, by testing two main hypotheses related to connectivity and episodic memory function in newly diagnosed patients with TLE.

In a previous study, we recently found slight cognitive deficits at the time of diagnosis in the same cohort of patients as utilized in the present study (26). Reduced scores on some of the executive function tests led us to suspect that extratemporal or network dysfunction might be present at an early stage. To expand on this, we addressed two key questions in the present study: (1) Are changes in parietofrontal connectivity present in the newly diagnosed patients with TLE? And (2) do newly diagnosed patients with TLE show reductions in the autobiographical episodic memory? To assess this, we employed EEG effective connectivity measures, which hitherto have been less utilized in research, despite having the advantage to be readily available for clinical samples and to provide good temporal resolution to capture connectivity. We chose the theta band activity, as this has been found to be central when it comes to studying the association between electrophysiology and cognition (27–30), while the relationship between memory and alpha-band activity is more uncertain (31) and is more sensitive to changes in arousal. We also implemented a memory test aimed at capturing experiential aspects of the episodic memory such as perceptual richness, remembered details, and vividness of the specific event (15, 32). We argue that such a measurement may represent a complementary perspective on episodic memory not captured by traditional list-learning tests. As our cohort consists of the newly diagnosed TLE, we minimize confounding variables present among the chronic patients (effects of recurrent seizures, medication, and surgery), and thus catch a glimpse of how TLE may manifest from its early onset.

## Materials and Methods

### Participants

The participants are part of an ongoing longitudinal study at the Epilepsy Unit, Department of Neurology, Oslo University Hospital Rikshospitalet, Oslo, Norway, in cooperation with the Department of Psychology, the University of Oslo. The patients have been enrolled from the neurological departments in the South-Eastern Health Region of Norway shortly after diagnosis. Controls were recruited by the patients, who were asked to bring a friend of the same sex and approximately the same age (±1 year) without epilepsy, to minimize differences in the socioeconomic factors. If the patients were not able to find a control, a control was recruited through advertisements at the Oslo University Hospital and the University of Oslo. These controls were also individually matched to the patients in age, gender, and level of education. The clinical and demographical characteristics of the patients are shown in Table 1.

**Table 1:** Demographical and clinical characteristics of patients with newly diagnosed, nonlesional temporal lobe epilepsy and healthy control subjects.

	**Patients**	**Control subjects**	***P*** -value^***^
	***n*** **= 21**	***n*** **= 21**	
**Age (years), mean (SD) [range]**	31.8 (11.7) [19–58]	31.7 (11.6) [19–58]	0.80
**Gender (male/female), n (%)**	7/14 (33/67)	7/14 (33/67)	
**Intelligence quotient (WASI-I), mean (SD) [range]**	113.5 (11.2) [74–127]	116.2 (9.5) [95–132]	0.41
Vocabulary	58.19 (5.41) [43–67]	60.14 (5.84) [47–71]	0.23
Matrix reasoning	56.81 (10.05) [20–66]	58.10 (6.49) [47–68]	0.67
**Dominance, n (%)**			0.15
Right-handed	20 (95)	17 (81)	
Left-handed	1 (5)	4 (19)	
**Seizure type, n (%)**			
Focal aware seizures	3 (14)		
Focal impaired awareness seizures	3 (14)		
Focal aware seizure and focal impaired awareness seizures	7 (33)		
Bilateral tonic-clonic seizures with focal onset	8 (38)		
Focal aware seizure and bilateral tonic-clonic seizures	3 (14)		
Focal impaired awareness seizure and bilateral tonic-clonic seizures	5 (24)		
**Use of antiseizure medication (ASM) in the study, n (%)**			
No	7 (33)		
Under titration	6 (29)		
Yes	8 (38)		
**Magnetic resonance imaging (3 Tesla) findings, n (%)**			0.75
Normal	9 (43)	8 (38)	
Abnormalities not associated with epilepsy^*^	12 (57)	13 (62)	
Mesial temporal sclerosis	0 (0)	0 (0)	
**25-channels electroencephalography findings, n (%)**			
Normal	5 (24)		
Abnormalities^**^	16 (76)		
Non-specific	10 (48)		
Abnormal theta rhythms	2 (10)		
Sharp waves	8 (38)		
Epileptiform			
Focal spike discharges	10 (48)		
**Self-reported mental health status, mean (SD) [range]**	67.81 (19.29) [20–92]	78.86 (11.67) [56–96]	0.04

*WASI-I, wechsler abbreviated scale of intelligence, first edition*.

* *Four patients and one control subject have several abnormalities. For details of these mainly clinically insignificant findings, see Bjørke et al. (26) for details*.

** *Four patients have both nonspecific and epileptiform abnormalities*.

*** *P-values are calculated using Pearson's chi-squared test [n (%)], t-test {mean (SD) [range]} or Kruskal-Wallis test [median (range)], as appropriate*.

Inclusion criteria were newly diagnosed (within 1 year) TLE, no structural brain lesions other than the mesial temporal sclerosis on MRI, and between 18 and 59 years of age. Since most recruited participants during inclusion were found to be nonlesional and no one appeared to have mesial temporal sclerosis, only patients with nonlesional TLE were included. The clinical diagnosis in each case was made by the same group of the experienced epileptologists, according to the seizure semiology, interictal EEG recordings, and qualitative assessment of structural MRI data according to ILAE definitions (see Table 1) (26). The MRI scans were acquired on a 3T whole-body scanner (Philips Ingenia®, Best, The Netherlands). The MRI protocol focused both on high-resolution images for the best possible visual characterization of anatomy and pathology [sagittal T1 volume, sagittal FLAIR volume, axial and coronal T2 (2 mm), and axial susceptibility-weighted imaging (SWI)], and also quantifiable measures including axial diffusion tension imaging (DTI) with calculated apparent diffusion coefficient (ADC) maps.

Although a diagnosis of TLE in the absence of ictal EEG recordings might be uncertain, the inclusion of ictal EEG recordings for the purposes of this study was not feasible because of the ethical reasons and cost-benefit judgments. Control subjects were individually matched to patients based on their sex, age, and education level. Exclusion criteria for both the patients and controls were known etiologies, such as tumors, infarctions, and malformations, in addition to intellectual disability, severe psychiatric disorder including depression and anxiety, progressive medical conditions, alcohol or drug abuse, and previous brain surgery.

The written informed consent was obtained from all the participants, and the study was approved by the Regional Committees for Medical and Health Research Ethics (reference number: 2013/855/REK sør-øst A).

### Study Procedure

Patients were examined by the experienced neurologists, neuropsychologists, and neurophysiologists at the Oslo University Hospital, Rikshospitalet, Oslo, Norway as part of the ProTLE study. ProTLE is a longitudinal study that aims to follow newly diagnosed patients with TLE and their matched controls for a period of 10 years (26). Patients arrived for a full day of testing at the hospital, participating in the clinical neurological assessment and neuropsychological testing, in addition to MRI and EEG. All the measurements were performed in separate sessions. Thus, EEG measurements do not reflect activity elicited by memory tasks but will rather be correlated with memory functions as part of the analysis.

### EEG Data Acquisition and Preprocessing

A 1 h 64-channel awake interictal EEG was performed on all the patients and controls with a Natus NicOne system with a C64 amplifier. No preprocessing was applied besides high- and low-pass filters in the amplifier at 0.5– 70 Hz, respectively. To minimize changes in arousal, participants were asked to rest with their eyes closed for the first 10 min, and with their eyes open for the next 50 min. The first 10 min constituted a recording resting patient with closed eyes. For the remaining 50 min, the participants were asked to read a book or watch a video to avoid falling asleep. The setup was used to have both a standard baseline recording and a relatively stable arousal level during the rest of the recording. The electrodes were placed in accordance with the 10-10 system by trained EEG technicians. The reference electrode was placed at CP1, and then re-referenced to the common average (33). Since our hypotheses were concerned with the parietofrontal network, only the P3, F3, P4, and F4 channels were used for the analysis in this study.

To reduce the influence of artifacts, the same procedures used by Schumacher et al. (34, 35) were implemented. By using the median instead of the arithmetic mean, the contribution of high-amplitude activities, main artifacts, is removed. This has been found to be as effective as manually discarding artifacts (35).

To assess effective connectivity between the brain regions of interest, the outflow was estimated by the directed transfer function (DTF) of theta activity (4–7 Hz) in the parietofrontal network. DTF was also used in order to avoid volume conduction issues, as it is found to be robust even in a low signal-to-noise ratio (36). Only the theta activity was assessed. The theta activity plays an important role in the temporal lobe. Fast activities are often phase-locked to this theta. Because of the dispersion effect, you would not expect direct connectivity measures in the gamma band between brain regions, probably only cross-frequency coupling with theta. The alpha activity is prone to large changes related to changes in the arousal level and activity.

The EEG data analysis was carried out by using the eConnectome (2.0) software [eConnectome: A MATLAB toolbox for mapping and imaging of brain functional connectivity (37)]. DTF was calculated for P3-F3 and P4-F4 channels, with a model order of 5. A total of four electrodes in the frontal and parietal regions were used in the calculations. Hence, the measured activity is constrained to these four electrodes. DTF was calculated for every 1 s segment of the raw data. All the combinations of electrodes calculated yield a total of 16 calculated connections for the full 1-h recording. The maximum value of each frequency in the theta band for each second was used. DTF was calculated for each second. The median of each channel outflow was used in the following computations. In each analysis, all the connections from the connection matrix that satisfied the criteria were used. For example, for the hemispheric symmetry connectivity, outflow to electrodes of the opposite side was calculated for each electrode and the median was calculated. The parietal regions were measured based on the P3 and P4 electrodes, and the frontal regions were based on the F3 and F4 electrodes (34, 38).

### Memory Tests

The episodic memory test is based on cued memory paradigms by Schacter et al. (19), and also a shortened, adapted, and translated version of The Memory Experiences Questionnaire, which measures the phenomenological characteristics of the episodic memories (15, 39). The test is administered digitally through Microsoft Powerpoint 2010, and the instructions are presented to the participants orally. The participants are presented with a cue word on the screen (e.g., vacation), and are asked to think of an event that they have experienced within the last 2 years that this word reminds them of. The event should be specific, lasting up to a few hours, within a day. They are given some time to think of an event and are then given 40 s to think about it with their eyes closed. Afterward, they are asked to fill in a questionnaire to describe their subjective experience of remembering. The questionnaire consists of 12 claims about the memory (e.g., “I saw the event through my own eyes”, “I could hear sounds”, “It felt like I was there again”), which the participants rate on a Likert scale of 1 (totally agree). All the items are included in a mean episodic memory score. Inter-item reliability (Cronbach's α) for this task has been shown to be acceptable (39).

### Statistical Analysis

The statistical analyses were carried out using RStudio Version 1.3.1093 (R Core Team, 2013. R: A language and environment for statistical computing. R Foundation for Statistical Computing, Vienna, Austria. URL http://www.R-project.org/). A one-way between-groups multivariate analyses of variance (MANOVA) was used to assess differences between the patients and controls on connectivity measurements, after checking the score distributions for normality and homogeneity of variance. Paired samples *t*-tests were used to assess differences in the effective connectivity between hemispheres within each group, and differences between patients and controls in episodic memory scores. Age and sex were controlled for by using a matched pairs design, and hence these variables were not included as independent variables in the analysis.

## Results

### Decreased Episodic Memory Function in Newly Diagnosed Patients With TLE

**Figure 1:**
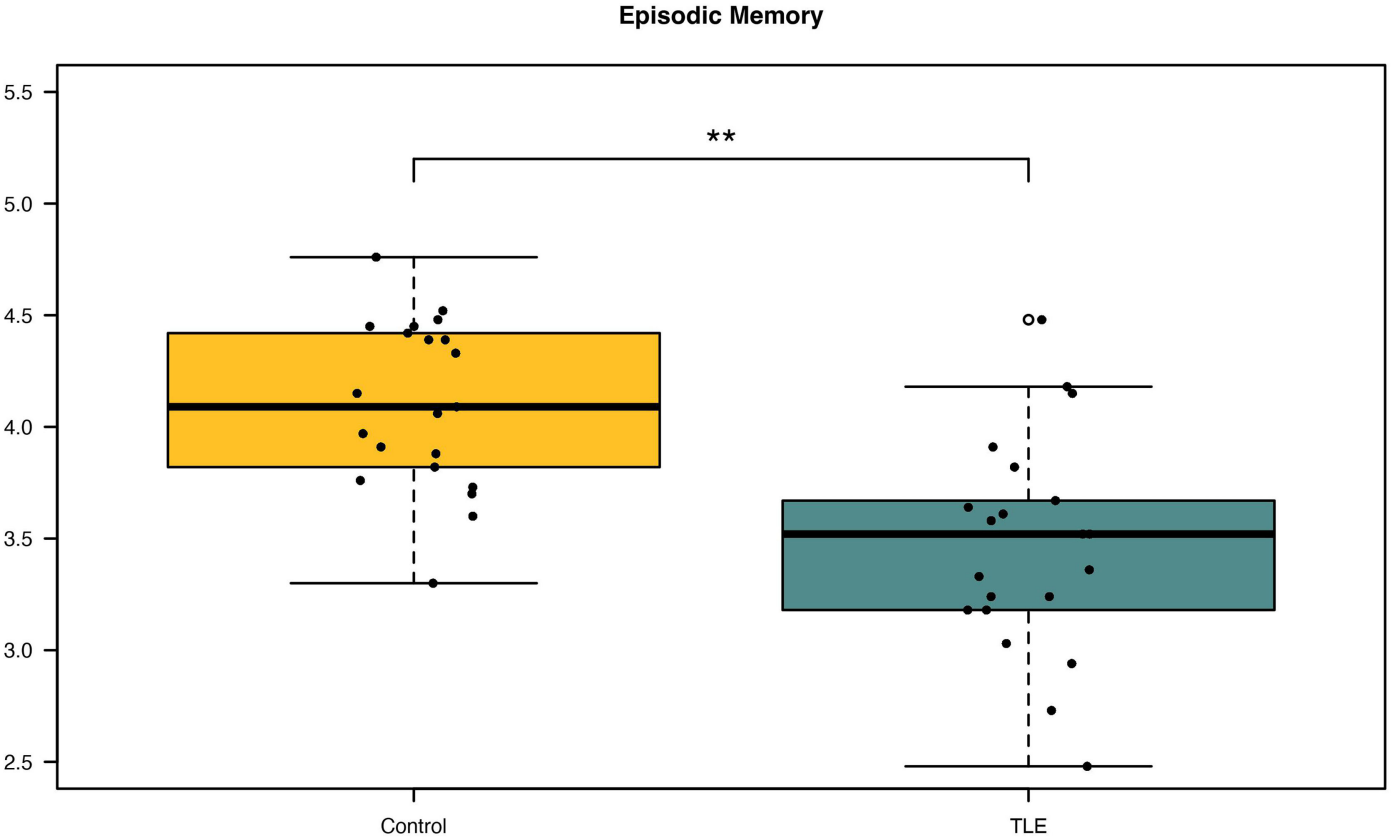
Differences in episodic memory scores between patients and controls. Patients with newly diagnosed temporal lobe epilepsy (TLE) were observed to have significantly decreased episodic memory scores compared with controls. **p < 0.001.

A paired *t*-test was done to determine the difference in episodic memory scores between patients with TLE and controls. Patients were found to have significantly lower episodic memory scores (M = 3.47, SD = 0.49, *p* ≤ 0.001) than controls (M = 4.10, SD = 0.37) (Figure 1). The mean difference in episodic memory score between the patients and controls were −0.64, with a 95% CI ranging from −0.89 to −0.38. The calculated Cohens d was found to be 1.13, indicating a large effect size. *Post-hoc* power analysis yielded a power of 0.9. Results are summarized in Table 2.

**Table 2:** Difference between TLE and controls on episodic memory, and between left and right parietofrontal connectivity in patients with TLE.

					***95% confidence interval***	
	**t**	**df**	**Mean of**	***P*** **-value**	**Lower**	**Upper**
			**differences**			
*Episodic*	−5.18	20	−0.64	<0.001^**^	−0.89	−0.38
*Connectivity*	−2.93	20	−0.11	0.008^*^	−0.19	−0.03

* *p < 0.05*,

** *p < 0.001*.

### Reduced Connectivity in the Right Parietofrontal Network in Newly Diagnosed TLE Patients Compared With Controls

**Figure 2:**
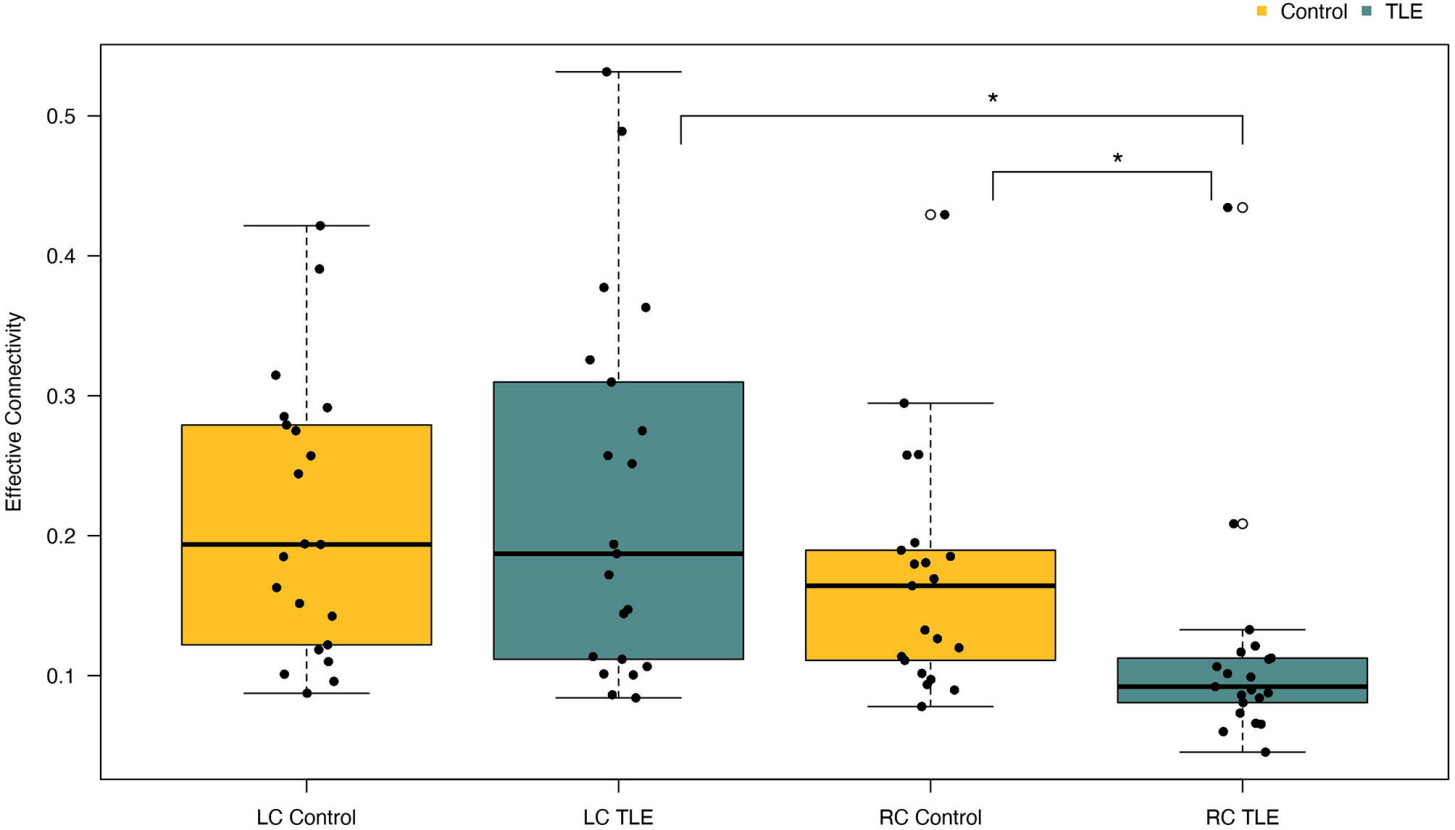
Differences in left and right parietofrontal connectivity between patients and controls. Patients with TLE had significantly reduced connectivity in the right hemisphere compared with controls. Furthermore, patients with TLE also had significantly reduced connectivity in their right hemisphere compared with their left. LC, Left connectivity; RC, Right connectivity. *p < 0.05.

A one-way between-groups MANOVA was performed to assess differences between patients and controls on the dependent variables left and right parietofrontal connectivity. There was no difference between the epilepsy and the control group on the combined dependent variables [F (2, 39) = 2,41, *p* = 0.10; Wilks' Lamda = 0.89; partial eta-squared = 0.11], indicating that there is no statistically significant difference between the TLE and the control groups on the combined dependent variables. When inspecting the results for each of the dependent variables, we observed a significant effect of right parietofrontal connectivity {F (1, 40) = 4.93, *p* = 0.03, 95% CI [−0.11; −0.005], partial eta-squared = 0.11}. Further inspection of the mean showed patients to have reduced right parietofrontal connectivity (M = 0.11, SD = 0.08) compared with controls (M = 0.17, SD = 0.09) (Figure 2). The partial eta-squared statistic (0.11) yielded a small effect size. There were no differences between patients and controls on left parietotemporal connectivity measurements. Results are summarized in Table 3.

**Table 3:** Differences between TLE and controls on connectivity variables (MANOVAs).

	**Df**	**Sum Sq**	**Mean Sq**	***F*** **-Value**	***P*** **-Value**
Left connectivity	1	<0.001	<0.001	0.16	0.69
Right connectivity	1	0.034	0.034	4.94	0.03^*^

* *p < 0.05. Df, degrees of freedom; Sum Sq, sum of squares; Mean Sq, mean squares*.

### Differences in Parietofrontal Connectivity Between Left and Right Hemisphere in Newly Diagnosed Patients With TLE vs. Controls

A paired *t*-test was conducted to determine the difference in effective connectivity between the left and right hemispheres (i.e., hemispheric connectivity asymmetry) in patients and controls. There was a statistically significant difference between effective connectivity in the right hemisphere (M = 0.11, SD = 0.08) compared with the left hemisphere [M = 0.23, SD = 0.13, *t* (20) = −2.93, *p* = 0.008] in patients. Inspection of the means shows patients to have stronger connectivity in the left hemisphere (Figure 2). The mean difference of connectivity between the left and right hemispheres of the patient group was 0.1121 with a 95% CI ranging from −0.19 to −0.03. Cohens d was calculated to be 0.640, indicating a medium effect size. *Post-hoc* power analysis revealed the power of the analysis to be 0.9. The controls also showed stronger connectivity in the left relative to the right hemisphere, although this effect was not significant (Figure 2). In summary, controls were observed to have higher connectivity than patients in the right parietofrontal network, whereas patients have higher connectivity in the left parietofrontal network. Furthermore, the difference in connectivity strength between the left and right parietofrontal networks is significantly larger among patients with TLE.

### Association Between Episodic Memory and Effective Connectivity

**Figure 3:**
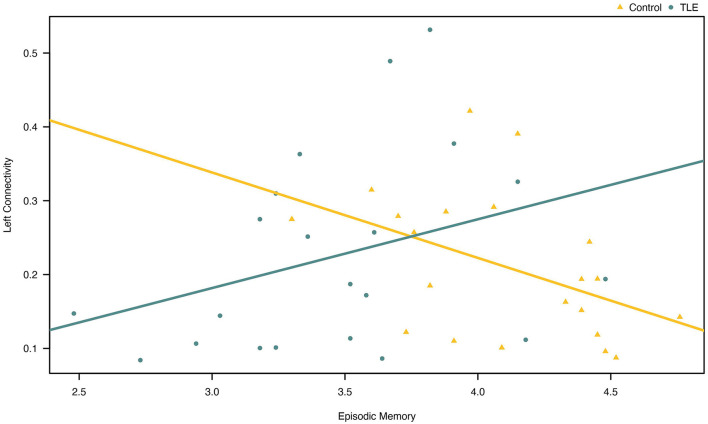
Association between episodic memory and effective connectivity in the left parietofrontal in patients and controls. Note that the correlation was only (barely) statistically significant for the controls (yellow line).

A Pearson product-moment correlation coefficient was calculated to explore the relationship between effective connectivity and episodic memory. We did not observe any linear relationships between episodic memory and left/right parietofrontal connectivity in patients with TLE. We did observe a statistically significant negative correlation between episodic memory scores and left parietofrontal connectivity in controls (*r* = −0.445, *N* = 21, *p* = 0.043) (Figure 3).

## Discussion

We observed a significant reduction in the episodic memory function in patients that were newly diagnosed with TLE. Patients with TLE were also observed to have decreased connectivity in their right parietofrontal network compared with controls, and they also had different symmetry in parietofrontal hemispheric connectivity from that observed in controls. There was no apparent relationship between episodic memory and connectivity measurements. Findings and interpretability are discussed in detail later.

### Effective Connectivity Changes in the Parietofrontal Network in Newly Diagnosed TLE

The results support the general notion that TLE is accompanied by alterations in the connectivity of the parietofrontal network, even in nonlesional newly diagnosed TLE. Specifically, we found that the patient group had reduced effective connectivity in the right parietofrontal channels relative to the controls (Figure 2). These findings are in agreement with the previous studies that have also found decreased connectivity in some parietal and DMN regions in patients with MTLE (6–8, 40). Also in line with our findings, reduced connectivity to the frontal areas has been reported in patients with MTLE (41, 42). To date, there exists no good explanation for these findings. One may speculate that it could be because of the structural degeneration caused by TLE that has spread to regions structurally connected to the MTLs—such as the parietal and frontal regions. Another possibility is that such changes reflect a secondary propagation network (3, 6, 41, 43).

A study by Zhang et al. (44) observed decreased connectivity in both ipsi- and contralateral DMN areas, such as the MTLs, inferior temporal cortex, and medial PFC, among patients with chronic TLE. They suggest that ipsilateral network alterations reflect impairment due to lesions caused by repetitive seizures, while decreased contralateral connectivity is caused by widespread functional impairment. Hence, it has been demonstrated that a focal lesion at a specific network node can lead to extensive changes in the remaining DMN. Several studies have found that epilepsy might lead to a decrease in connectivity over time (6, 8, 40, 44). If this is true, it is possible that the full detrimental effects of epilepsy have not set in with this study cohort. However, the question of whether TLE (or other epilepsy forms) have to be seen as progressive disorders does not yet yield any clear-cut answers, and researchers have found evidence both for and against time-dependent deterioration of brain networks (45).

The finding that patients exhibited differences between left and right connectivity patterns leads us to speculate that the effects of TLE has spread from the MTLs to the parietofrontal network. It is thinkable that stronger functional integrity makes the left hemisphere more resistant to damage, and thus, we only observed reduced connectivity in the right hemisphere (46). Even more likely, it is possible that seizure lateralization influences the observed connectivity alterations. It is well-established that the MTL ipsilateral to the EZ suffers from the most extensive damage (1, 4, 6, 8). Unfortunately, as our cohort consists of patients with nonlesional TLE, we cannot ascertain the origin of epileptic activity, and defining clinical signs of lateralization in the very early stage of the disorder is not reliable enough to use for further analyses. Therefore, this finding must be considered observational and cannot be interpreted in the context of focus lateralization.

### Phenomenological Aspects of Episodic Memory

The finding of reduced episodic memory is in accordance with the previous studies, which have reported decreased episodic memory function in chronic TLE (47–50). As far as we know, no other study has explored experiential aspects of episodic memory functions in newly diagnosed patients with TLE. Importantly, instead of only using list-learning tasks as measures of verbal and visual episodic memory, this study aimed to measure autobiographical episodic memory by assessing the subjective memory experience of the participants. This study, therefore, finds evidence that aspects of autobiographical episodic memory, such as vividness, perceptual richness, and remembered details are impaired in the newly diagnosed patients with TLE.

### Episodic Memory as a Network

We hypothesized that the parietofrontal network is important for episodic memory retrieval because it plays a crucial role in the reconstruction process of personal memories. Based on the existing literature [mostly functional MRI (fMRI) studies], increased connectivity tends to be associated with increased functionality—i.e., better performance on cognitive tests (5, 51, 52), whereas decreased connectivity is considered to be caused by disruption of neural connections and associated with cognitive decline (52, 53). However, per this study, we did not find a significant association between episodic memory scores and connectivity in patients, and only observed a significant correlation between episodic memory and left parieto-frontal connectivity in the controls. Here, we present some possible causes for the findings of this study.

Although some studies using fMRI have found decreased connectivity in patients with TLE (5, 8, 41), other studies using EEG and magnetoencephalography have found the opposite pattern (5, 6, 54). Such contrasting findings could be linked to the different physiological underpinnings of each method. Specifically, it is possible that epilepsy has led to hyperexcitable links between structures, constituting the epileptogenic network. Such epileptiform activity can contribute to a larger EEG signal. Conversely, studies have found that excessive electrical activity can lead to metabolic abnormalities both in the EZ and strongly connected regions (55). Such metabolic abnormalities will in turn lead to a weaker BOLD response, which will be interpreted as decreased connectivity (5, 55, 56). This entails that although increased connectivity as measured by fMRI is commonly interpreted as enhanced functionality, this might not hold true in EEG measures of epileptic patients. Rather, increased neuronal activity in epilepsy is likely to reflect pathological malfunction, possibly representing an epileptogenic and/or a seizure propagation network (54). It is also important to note that although EEG and fMRI findings are not always contrasting, comparative analyses should be interpreted with caution (2).

We cannot be certain that the theta band is optimal for capturing the DMN, and there is a possibility that we would have obtained different results if we had adopted another frequency band. Previous studies have also found correlations between DMN activity and theta- (57), alpha- (58–60), beta- (59, 60), and gamma-power band (59, 61). More research is warranted to better understand the association between BOLD signals and neuronal activity in order to better map the electrophysiological signals underlying DMN activation (58, 60, 62).

Another critical issue in connectivity studies is related to the choice of the network to be measured. This concern may also be raised in this study as others often related the DMN as a measure for the episodic memory. By using EEG, we might have measured other aspects of brain networks than what has been reported by studies using fMRI. With this in mind, a direct comparison of our results to findings from fMRI studies investigating the DMN should be done cautiously.

Both EEG connectivity and episodic memory disturbances point to TLE affecting the brain beyond the medial temporal lobe area, supporting several findings from the last years highlighting TLE as a network disorder (2, 4, 41, 44, 63–65). The lack of a relationship between EEG connectivity and episodic memory scores in patients was unexpected, but this topic needs further investigation.

### Limitations

Studying autobiographical episodic memory has the advantage of including general, network-based functions, and might be more relevant for clinical care. A potential shortcoming with this method is that it depends on subjective ratings and may thus reflect a bias in terms of disease attributions of the patients. This method is being increasingly used in research on various clinical populations, and more studies are needed, also in the field of epilepsy.

It is likely that seizure lateralization influences connectivity networks in patients with TLE, also if they are newly diagnosed. It is well established that the MTL ipsilateral to the EZ suffers from the most extensive damage (4, 6, 8). Unfortunately, because our patients are in the early stages of epilepsy, and video-EEG recordings of seizures were unobtainable, it was not possible to ascertain seizure lateralization in this study. Nonetheless, there is a possibility that like the MTLs, connectivity in the parietofrontal regions is affected differently depending on the lateralization of the seizure focus.

A practical limitation of this study is that participants might not have been resting entirely throughout the session. Studies have shown that the most important consideration to ensure DMN activity is a low-cognitive load (66). If reading or watching a video constitutes a high-cognitive load for the participants, it may have caused task-related activity and DMN deactivation. Nevertheless, we chose to use the entire hour of the EEG recording, and not only the 10 min of closed eyes resting-state measurement, because visual inspection of the 10-min data revealed unrest and arousal in patients. This arousal stabilized with increasing acquisition time. We, therefore, consider the entire measurement to constitute a more robust measure than the 10 min alone.

At last, we want to mention our relatively small sample size as a limitation. We hope that this study is able to contribute as an explorative study on connectivity and episodic memory alternations in TLE despite the small sample size, and hope it will be repeated in the larger samples to better understand the underlying phenomena.

## Conclusion

In this study, we have observed alterations in both connectivity and in episodic memory in patients with newly diagnosed TLE. These two features, each one individually, support the notion that TLE might affect extratemporal networks, and that these features are already present in the early course of epilepsy. Future studies may overcome technical and methodological limitations and investigate whether there is a progressive change in the connectivity patterns, memory functions, and possible associations between these in TLE. A better understanding of connectivity abnormalities may have clinical implications and could serve as an effective diagnostic tool for epilepsy in the future and might also aid in decision making for personalized treatment strategies. Moreover, a better understanding of the association between connectivity and memory may shed light on the complex neuronal underpinnings of episodic memory—a remarkable and distinctive capability of human beings.

## Data Availability Statement

The raw data supporting the conclusions of this article will be made available by the authors, without undue reservation.

## Ethics Statement

The studies involving human participants were reviewed and approved by the Regional Committee for Medical and Health Research Ethics (REC) (reference number: 2013/855/REK sør-øst A). The patients/participants provided their written informed consent to participate in this study.

## Author Contributions

ABj, YØ, PL, and ABa: manuscript writing, preparation and revision. ABa: statistical analysis. ABj, ABa, MN, and YØ: data collection. PL: data preparation. PL, KO, and YØ: data analysis. ABj, YØ, PL, KO, ET, KH, and ABa: study design. ET and KH: manuscript preparation and revision. KO: manuscript revision. All authors read and approved the final manuscript.

## Funding

This project has been made possible by Dam Foundation, Norway and supported by the Norwegian branch of the International Bureau for Epilepsy (IBE).

## Conflict of Interest

The authors declare that the research was conducted in the absence of any commercial or financial relationships that could be construed as a potential conflict of interest.

## Publisher's Note

All claims expressed in this article are solely those of the authors and do not necessarily represent those of their affiliated organizations, or those of the publisher, the editors and the reviewers. Any product that may be evaluated in this article, or claim that may be made by its manufacturer, is not guaranteed or endorsed by the publisher.

## Nomenclature

### Resource Identification Initiative

To take part in the Resource Identification Initiative, please use the corresponding catalog number and RRID in this manuscript. For more information about the project and for steps on how to search for an RRID, please click here.

### Life Science Identifiers

Life Science Identifiers (LSIDs) for ZOOBANK registered names or nomenclatural acts should be listed in the manuscript before the keywords with the following format:

urn:lsid:<Authority>:<Namespace>:<ObjectID>[:<Version>] For more information on LSIDs please see the Inclusion of Zoological Nomenclature section of the guidelines.
